# Upland rice varietal mixtures in Madagascar: evaluating the effects of varietal interaction on crop performance

**DOI:** 10.3389/fpls.2023.1266704

**Published:** 2023-11-20

**Authors:** Koloina Rahajaharilaza, Bertrand Muller, Cyrille Violle, Kirsten vom Brocke, Ramavovololona, Jean Benoît Morel, Elsa Balini, Florian Fort

**Affiliations:** ^1^ University of Antananarivo, Faculty of Sciences, Antananarivo, Madagascar; ^2^ CIRAD, UMR AGAP Institut, Montpellier, France; ^3^ Dispositif en Partenariat Système de Production d’Altitudes Durable, CIRAD, Antsirabe, Madagascar; ^4^ UMR AGAP Institut, Univ Montpellier, CIRAD, INRAE, Institut Agro, Montpellier, France; ^5^ CEFE, Univ Montpellier, CNRS, EPHE, IRD, Montpellier, France; ^6^ PHIM Plant Health Institute, Université de Montpellier, INRAE, CIRAD, Institut Agro, IRD, Montpellier, France; ^7^ CEFE, Univ. Montpellier, L’Institut Agro, CNRS, EPHE, IRD, Montpellier, France

**Keywords:** crop management, upland rice, biodiversity, varietal mixtures, ecosystem functioning and productivity, disease resistance

## Abstract

**Introduction:**

Rice plays a critical role in human livelihoods and food security. However, its cultivation requires inputs that are not accessible to all farming communities and can have negative effects on ecosystems. simultaneously, ecological research demonstrates that biodiversity management within fields contributes to ecosystem functioning.

**Methods:**

This study aims to evaluate the mixture effect of four functionally distinct rice varieties in terms of characteristics and agronomic performance and their spatial arrangement on the upland rice performance in the highlands of Madagascar. The study was conducted during the 2021-2022 rainfall season at two close sites in Madagascar. Both site differ from each other’s in soil properties and soil fertility management. The experimental design at each site included three modalities: i) plot composition, i.e., pure stand or binary mixture; ii) the balance between the varieties within a mixture; iii) and for the balanced mixture (50% of each variety), the spatial arrangement, i.e., row or checkerboard patterns. Data were collected on yields (grain and biomass), and resistance to Striga asiatica infestation, Pyricularia oryzea and bacterial leaf blight (BLB) caused by Xanthomonas oryzae-pv from each plot.

**Results and discussion:**

Varietal mixtures produced significantly higher grain and biomass yields, and significantly lower incidence of Pyricularia oryzea compared to pure stands. No significant differences were observed for BLB and striga infestation. These effects were influenced by site fertility, the less fertilized site showed stronger mixture effects with greater gains in grain yield (60%) and biomass yield (42%). The most unbalanced repartition (75% and 25% of each variety) showed the greatest mixture effect for grain yield at both sites, with a strong impact of the varietal identity within the plot. The mixture was most effective when EARLY_MUTANT_IAC_165 constituted 75% of the density associated with other varieties at 25% density. The assessment of the net effect ratio of disease, an index evaluating the mixture effect in disease reduction, indicated improved disease resistance in mixtures, regardless of site conditions. Our study in limited environments suggests that varietal mixtures can enhance rice productivity, especially in low-input situations. Further research is needed to understand the ecological mechanisms behind the positive mixture effect.

## 1 Introduction

Rice is a staple food for over half of the world’s population, thus making it an essential crop in ensuring food security (Breumier et al., 2018; Gérardeaux et al., 2021; OECD-FAO, 2023). In Madagascar, like in many African countries, rice is grown mainly in upland and lowland areas (Radanielina et al., 2013). However, its cultivation is challenged by high incidence of pests and diseases, soil degradation, and unpredictable weather conditions (Balasubramanian et al., 2007; Gerardeaux et al., 2012; Raboin et al., 2014). Conventional agricultural practices used for growing monoculture or the cultivation of pure stands of some resilient varieties like CHOMRRHONG DHAN and NERICA have come under scrutiny due to their negative environmental impacts, including the loss of biodiversity, water pollution, and soil degradation (Malézieux, 2012). These constraints collectively contribute to increase vulnerability of crops to biotic and abiotic stresses (Raboin et al., 2013) and pose a significant challenge in sustaining upland rice production without detrimental environmental impacts.

Genotypic diversity has a positive effect on the functioning of ecosystems and enhance diverse ecological services (Naeem et al., 2012; Boero, 2015; Turnbull et al., 2016; Panda et al., 2021). Moreover, research in functional and community ecology has been carried out to understand the mechanisms underlying biodiversity effect on ecosystems (Cardinale et al., 2012; Jucker and Coomes, 2012). Ecologists have developed and tested various hypotheses on the mechanisms to understand the link between community functioning and diversity (Cardinale et al., 2011; Miele et al., 2018). Two major mechanisms have been identified: the complementarity effect and the selection effect (Loreau and Hector, 2001; Wang et al., 2021). These mechanisms are linked to the fact that different species or genotypes fulfil different functions that contributes to the functioning of ecosystems (Yachi and Loreau, 1999; Hooper et al., 2005). The complementary effect is defined as the positive effect of increasing diversity due to the efficient partitioning of the available resources or positive interaction among species. The selection effect is due to the dominance of one or several species or genotypes well-adapted to the environmental conditions faced by the community (Loreau and Hector, 2001; Wang et al., 2021). Cardinale et al. (2007) demonstrate that, although mixtures generally produce more biomass than monocultures, only a few outperform the best-performing monoculture. This emphasizes the significant role of species complementarity. These findings have resonated with agronomic sciences, which are increasingly exploring the role of biodiversity in agroecosystem functionality (Altieri, 1999; Feledyn-Szewczyk et al., 2016; Barot et al., 2017). A wide range of studies has shown that introducing diversity into a field can lead to increased productivity, whether through inter-specific diversity (Cardinale et al., 2002; Gaudio et al., 2021) or intra-specific diversity (Snyder et al., 2020; Wuest et al., 2021). Li et al. (2023) further demonstrated that while intercropping may yield a diverse range of crop products, it may slightly lag in grain or calorie yield compared to the best monoculture. However, its protein yield, especially with maize/legume combinations, often matches or surpasses monocultures. Such crop mixtures also mitigate the negative effects of disease propagation within the field (Fréville et al., 2022). However, the mechanisms behind these effects remain, all too often, unexplored in agroecosystems, as does their interaction with the crop management system.

One way to increase the within field biodiversity is the use of varietal mixtures. The potential benefits of mixtures, including increased productivity (Creissen et al., 2016), stability, and sustainability (Smithson and Lenné, 1996; Reiss and Drinkwater, 2018) are well-established and this practice is increasingly widespread in upland rice production (Smithson and Lenné, 1996; Revilla-Molina, 2009). Yet, varietal mixtures have been shown to reduce the risk of pest and disease outbreaks (Singh et al., 2016; Zhu et al., 2000) as well as yield losses due to extreme weather events (Creissen et al., 2016). Additionally, mixtures can increase crop resilience, improve nitrogen cycling, and reduce soil erosion (Cardinale et al., 2002; McAlvay et al., 2022). Consequently, the use of varietal mixtures can reduce the need for chemical inputs, resulting in economic and environmental benefits (Borg et al., 2018; Snyder et al., 2021).

However, the effectiveness of varietal mixtures may depend on their growth conditions including both, spatial organization of the mixture and fertility of the field. Studies have shown that a crop’s mixture organisation can have a significant impact on crop performance and can influence, in different ways, vegetative and reproductive biomass production in favour of grain yield (Gaudio et al., 2021; C. Li et al., 2020), as well as disease control (Newton and Skelsey, 2023). As the mixture effect is determined by plant-plant interactions, we hypothesized that the proportion and spatial organization of the mixture could have a strong effect on the agronomic traits. Based on an extensive literature review, Reiss and Drinkwater (2018) demonstrated that different soil fertility components can influence mixture effects and determine the relevance of the mixture in comparison to pure stand. They concluded that the benefits of mixtures were more prevalent in low-fertile soils than in relatively more fertile soils. While prior research has provided valuable insights, there remains an ongoing need for a comprehensive understanding of the complex relationships between mixture performance, their spatial arrangement, and the environmental conditions they encounter. These critical gaps in the existing literature serve as the foundation for our research questions in this.

The main objective of the study was to assess the effect of varietal mixture of upland rice, their proportion and spatial arrangements on agronomical performance of yield and disease resistance. We then aimed at these three sub-objectives: i) assess the relevance in terms of yield and disease resistance of growing upland rice varieties in mixtures compared to monoculture, and whether the mixture effect is influenced by the environment; ii) test whether the spatial arrangement of the binary mixtures, i.e., both the proportion of each variety as well as their organisations, has an impact on the mixture effect; and iii) estimate if some genotypes are more efficient than others to build effective mixtures. To reach these objectives, we conducted field experiments in the highlands of Madagascar with four functionally contrasted upland rice varieties grown in mixture and pure stand. Two experimental sites were established with two different levels of fertilization. To assess the influence of environmental limitations on the performance of the varietal mixtures, we considered constraints such as the weed *Striga asiatica*, as well as the diseases *Pyricularia oryzea*, and *Bacterial Leaf Blight* (BLB).

## 2 Materials and methods

### 2.1 Study site and trial management

In order to compare the performance of upland rice under two contrasting growing conditions, the same experiment was conducted simultaneously at two relatively close sites both featuring “Ferralsol” soil type with differing properties (**Table S1**) and distinguished soil fertility management. These sites are located in the western part of theVakinankaratra region, within the central highlands of Madagascar. The first site, Ankazomiriotra (in the following referred to as “Site ANK”), is located at an altitude of 1128m, (19°40’00.92”S, 46°32’11.49”E). The second site, Ivory (referred to as “Site IVO”), is situated at an altitude of 950m (19°33’21,7”S 46°25’58,4”E). These two sites are approximately 16km apart from each other.

During the growing season, which extended from October 2021 to May 2022, both sites experienced very similar meteorological conditions. The average monthly rainfall was 182mm at Site ANK and 183mm at Site IVO. The average temperature throughout the experiment was 25.4°C, with a minimum temperature of 18°C and a maximum temperature of 37°C at both locations.

The soils at both sites are classified as Ferralsol (FAO, 2014), known for their low nitrogen, carbon, and available phosphorus content. The soil at the ANK site had a higher percentage of clay in the upper horizons (0-10cm, 10-20cm, and 20-30cm) compared to the IVO site. In contrast, the IVO site had higher percentages of fine silts and coarse silts in these horizons (**Table S1**). According to the World Reference Base for Soil Resources (2014), the ANK site has a “Clay loam” soil, while the IVO site has “Silty Clay Loam” soil. The percentage of fine sand was similar at both sites ( ± 9%), while the percentage of coarse sand was higher at Site ANK (34%) than at Site IVO (20%). The chemical properties of the soils were also analysed and showed that the pH was slightly higher at Site IVO (mean = 5.0) than at Site ANK (mean = 4.8) in all three horizons. The organic carbon content was greater in the topsoil (0-10cm) at Site ANK (21.5g.kg^-1^) compared to Site IVO (17.6g.kg^-1^), as was the case for the total nitrogen content (1.7g.kg^-1^ and 1.4g.kg^-1^, respectively). The assimilable phosphorus content was lower at Site ANK (mean =5.0mg.kg^-1^) compared to Site IVO (mean =6.9mg.kg^-1^) in all three horizons. Soil at the ANK site is rich in organic matter (20.9 g.kg^-1^) compared to soil at the IVO site (16.9 g.kg^-1^). Detailed information about soil characteristics and properties is available in **Table S1**. The differences in soil chemical properties may be influenced by the recent history of each field. The IVO site was previously cultivated with maize for three seasons, using chemical fertilizer, while the ANK site was previously cultivated with *Mucuna puriens*, a leguminous cover crop, after being left fallow for two years. Given this history, we implemented a more intensive cropping management approach at the IVO site, involving higher fertilizer application.

We conducted this experiment on two sites with different levels of fertilisation. At ANK site, an initial application of 5t DM.ha^-1^ of cattle manure resulted in an input of 31.2 kg.ha^-1^ of nitrogen (N) and 5.6 kg.ha^-1^ of phosphorus (P_2_O_5_). Subsequently, an application of 50kg.ha^-1^ urea further enriched nitrogen levels by 23kg.ha^-1^. In the case of IVO site, an initial application of 5t DM.ha^-1^ of cattle manure introduced 52.35 kg.ha^-1^ of N and 11.25 kg.ha^-1^ of P_2_O_5_. Dual applications of NPK (11:22:16) “Vano be” played a pivotal role in fertilisation. The first application at sowing time (50kg.ha^-1^), contributed 5.5kg.ha^-1^ of N, 11kg.ha^-1^ of P_2_O_5_, and 8 kg.ha^-1^ of potassium (K_2_O). The subsequent booting stage application (100kg.ha^-1^) added 11kg.ha^-1^ of N, 22 kg.ha^-1^ of P_2_O_5_, and 16kg.ha^-1^ of K_2_O. A 23kg.ha^-1^ of N by urea was also applied at this stage with the second application of NPK “Vano be”. As a result, the site ANK was fertilized with 54.2kg.ha^-1^ of N, 5.6kg.ha^-1^ of P_2_O_5_, and no amount of K_2_O. The site IVO received 91.85kg.ha^-1^ of N, 44.25kg.ha^-1^ of P_2_O_5_ and 24kg.ha^-1^ of K_2_O.

### 2.2 Plant material

Our aim was to work with varieties that shared similar growing cycle length but exhibited a diversity of morphological traits and resistance to *Pyricularia oryzea* (Zhang et al., 2016; Qi et al., 2019) in order to evaluate the effects of their mixtures under contrasting field conditions. Four upland rice varieties were selected for the experiment: EARLY MUTANT IAC 165 (A), FOFIFA 152 (B), FOFIFA 154 (C), and DOURADO PRECOCE (D). These varieties displayed significant differences in terms of phenotypic traits (**Table S2**), agronomic performance, and disease resistance, as indicated in a previous study (**Table S2**) (Rahajaharilaza, manuscript in preparation). Specifically, EARLY MUTANT IAC 165 and DOURADO PRECOCE are taller than FOFIFA 152 and FOFIFA 154. Additionally, according to the previous study, variety A proved to be the most productive, while D exhibited the highest sensitivity to *Pyricularia oryzea* (Zhang et al., 2016; Qi et al., 2019).

### 2.3 Experimental design

**Figure 1:**
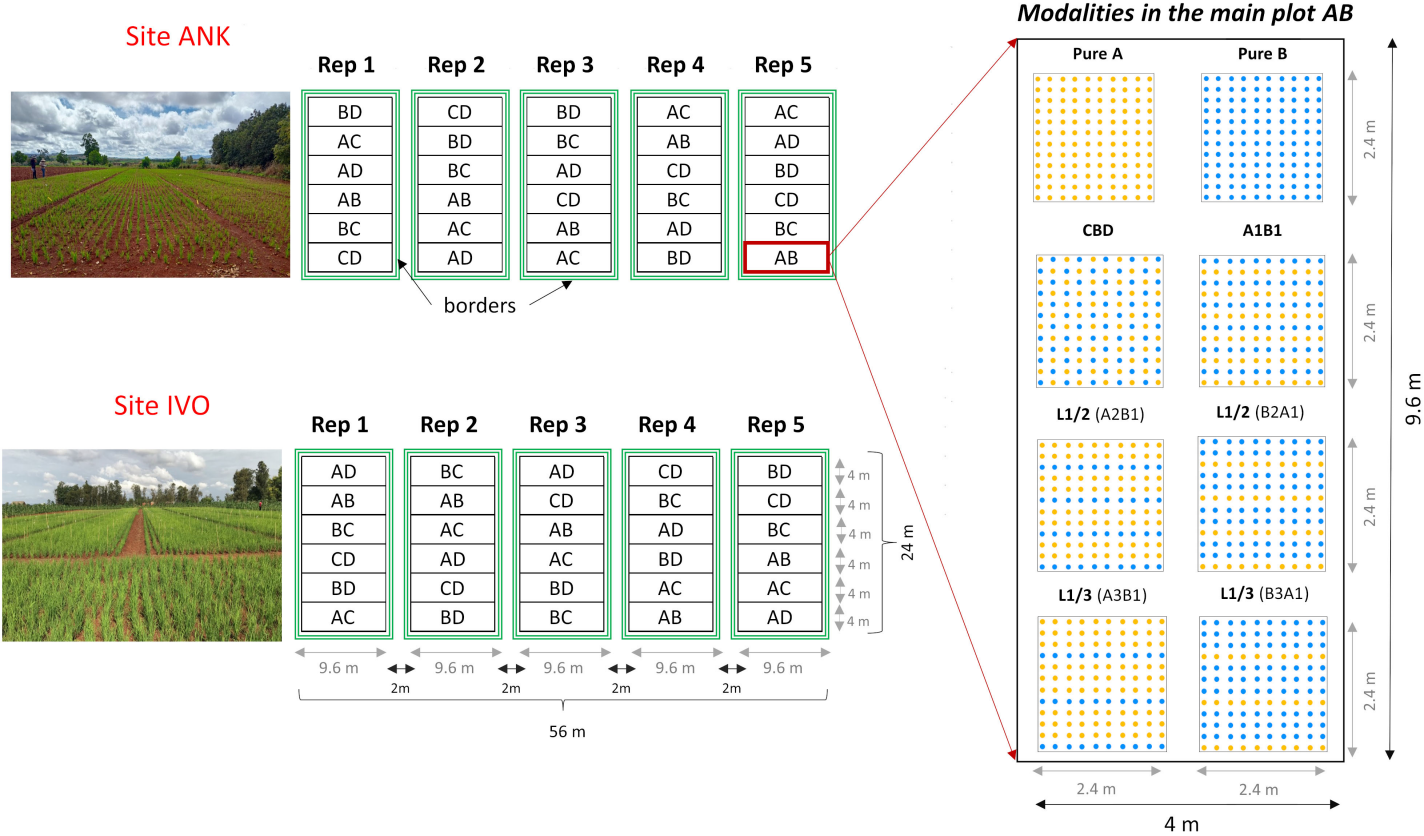
Split-plot experimental design with six main plots containing six binary mixtures, conducted in two experimental sites with five replications (blocks). Each block contains 48 microplots of 4.48 m². For the unbalanced repartition L1/2 the mixture A2B1 is composed by 67 percent of the variety A (EARLY MUTANT IAC_165) and 33 percent of the variety B (FOFIFA 152) and the mixture of B2A1 composed by 67% of the variety B and 33 percent of the variety A. In the same way for the repartition L1/3 the mixture A3B1 is composed by 75 percent of the variety A and 33 percent of the variety B and it is the opposite for the mixture B3A1. 'A' stands for EARLY MUTANT IAC 25; 'B' stands for FOFIFA 152; 'C' stands for FOFIFA 154; and 'D' stands for DOURADO PRECOCE.

The four selected genotypes were cultivated both in pure stands and mixed in pairs, resulting six binary mixtures. Each mixture was tested with four different arrangements of the hills of the two varieties: two balanced repartitions, with 50 percent of each variety, considering: i) alternating rows (referred to as L1/1) and ii) checkerboard pattern (CBD); iii) an unbalanced repartition, with two-thirds of the plants from one variety and one-third from the other one (67-33), obtained by alternating one in two rows (L1/2); and iv) a very unbalanced treatment, with three-quarters of one variety and one quarter of the other one (75-25), obtained by alternating one in three rows (L1/3). The mixtures were named using the codes of the varieties that composed them, considering first the most abundant one. For instance, the mixture composed of 67 percent of variety A and 33 percent of the variety B was named A2B1, with the first letter representing the most abundant variety in the mixture (see **Table 1**). In both sites, the experimental design was structured as a split plot with five complete blocks (repetitions). Each main plot represented a specific binary mixture as the main factor, with the spatial arrangement as the subplot factor (**Figure 1**). Randomization was implemented at two levels: first, by randomizing the order of the six combinations within each block, and second, by randomizing the eight modalities within each combination. This design was chosen to ensure a robust and reliable comparison of the treatments, with a primary focus on the variety effect. Each elementary plot (subplot) had a length of 2.4m and a width of 2m, resulting in a total area of 4.8m² which included 120 hills spaced at a distance of 20cm from each other (**Figure 1**).

**Table 1:** Treatments studied along a variety association taking the main plot EARLY_MUTANT_IAC_165 (A) and FOFIFA 152 (B) as an example in a split plot design experimental.

Main factor (Main plot)	Sub-plot factor	Variety associated	Descriptions
AB	Pure A	***Pure A***	Pure stand of variety EARLY_MUTANT_IAC_165 (A)
AB	Pure B	***Pure B***	Pure stand of variety FOFIFA 152 (B)
AB	CBD	***CBD***	Checkboard pattern of A (50%) and B (50%)
AB	L1/1	***A1B1***	Equal proportion of A (50%) and B (50%) in alternating rows one by one
AB	L1/2	***B2A1***	One row of variety A (37%) and two rows of variety B (63%)
AB	L1/2	***A2B1***	Two rows of variety A (63%) and one row of variety B (37%)
AB	L1/3	***B3A1***	One row of variety A (25%) and three rows of variety B (75%)
AB	L1/3	***A3B1***	Three rows of variety A (75%) and one row of variety B (25%)

Both pure stands and mixtures were managed in the same way. At sowing,six to eight seeds (of a single variety) were placed in small holes about 3cm deep covered with a small amount of soil, this is what we call “hill”. The cattle manure, and the mineral fertiliser in IVO, were added to these holes, before covering the planting holes with small amounts of soil. These planted hills were considered as individual plants during our study. The crop depended entirely on rainwater as no irrigation was provided. Weeding was carried out manually three times in all plots to facilitate optimal growth, specifically two weeks after sowing, at the start and at the end of tillering.

### 2.4 Plant measurements

Throughout the experiment, striga infestation, *Pyricularia oryzea* (Zhang et al., 2016; Qi et al., 2019) and Bacterial leaf Blight (BLB) caused by *Xanthomonas oryzae pv. oryzae (Xoo)* incidence (Tall et al., 2022) were recorded. The number of striga plants and their biomass were noted before each weeding operation, which occurred three times during the experiment. *Pyricularia oryzea* on leaves was assessed during flowering stage, while *Pyricularia oryzea* infection on panicles was noted during the maturity stage. Six hills per variety were selected on the diagonal of each plot to quantify *Pyricularia oryzea*. Two fundamental evaluation were made for *Pyricularia oryzea* infection: disease incidence and disease severity. Incidence referred to the proportion of infected tillers or panicles out of the total tillers/panicles on a hill and was typically expressed as a percentage. Severity characterized the extent of infestation and referred to the degree of infestation within individual infected tillers. We calculated the number of lesions on each leaf per infected tiller to represent disease severity. Considering both disease incidence and severity simultaneously provides a comprehensive understanding of the infection’s impact, allowing for a more nuanced assessment of the disease’s effects on the experiment. At the IVO site, measurements for BLB were also taken by counting the number of diseased hills per plot without identifying the variety involved.

To assess the phenotypic characteristics of each variety in both pure and mixture stands, five hills per variety in the middle of the plot were selected. The duration of the vegetative phases of these hills and the time to flowering was monitored. After the plants had bloomed, plant height was measured, and the number of leaves and stems in these five hills was recorded.

Harvesting was conducted plot by plot, by first removing all border hills in order to prevent contaminations and errors. Then, we gathered all the hills of the first variety of the mixture, and concluded with the hills of the second variety. Notably, our analysis of yield separated the grain and vegetative aboveground biomass components, or straw. For of grain yield, we carefully separated the grains from the panicles. In the case of vegetative aboveground biomass yield, the procedure involved isolating the straw without grain and panicle. Sample grain and vegetative biomass dry weights were established by drying subsamples for 48 hours at 60C°.

### 2.5 Relative productivity of mixture

As presented in equation 1 we calculated the relative yield total (RYT) for each plot. This measure is obtained by adding up the relative yield of each variety in the mixture (Hooper and Dukes, 2003; Weigelt et al., 2021):


(Eq 1) $$RYT\;\left(ij\right)=\frac{Y_{mix\left(i\right)}}{\;Y_{pure\left(i\right)}}+\;\frac{Y_{mix\left(j\right)}}{\;Y_{pure\left(j\right)}}\;$$

Where Y_mix(i)_ is the yield (grain or biomass) of variety (i) in the mixture and Y_pure(i)_ is the yield (grain or biomass) of variety (i) in pure stand in the same main-plot. A RYT value greater than 1 indicates a global positive effect on yield in the mixture compared to what could be expected based on the performances of its components in pure stands. RYT was calculated per main plot within a block to account for the block effect. The pure stand yields are, therefore, the average of the yields per block. The calculation of Relative Yield Total (RYT) shares a common underlying principle with the Land Equivalent Ratio (LER). Both assess the advantages of intercropping by comparing combined yields or resource utilization in varietal mixtures with those achievable in separate pure stands (De Wit, 1960; Mead and Willey, 1980).

### 2.6 Disease resistance of mixture

To assess resistance to *Pyricularia oryzea*, the net effect ratio of disease (NERDI) was calculated for the disease intensity (DI) (Cardinale et al., 2007; Li et al., 2023). This intensity was separated into disease incidence and severity.The NERDI measures the difference between the actual disease infection and the expected mixture infection based on pure crop infection and the variety proportions in the mixture. Unlike measuring reduction, our index calculates the sensitivity ratio of disease in mixtures versus pure stands within separate plots. Combining these relative indices involves aggregating the relative index of each variety in mixtures and comparing it to the average when that variety is in a pure stand within the mixed plots. A NERDI value above 1 indicates heightened disease in mixtures, while a value below 1 signifies the efficacy of mixtures in disease reduction.

The following formula was used to calculate the net effect ratio of disease:


(Eq 2) $$NERDI\;\left(ij\right)=\frac{DI_{observed}}{DI_{expected}}+\frac{p_{i}DI_{mix\left(i\right)}+p_{j}DI_{mix\left(j\right)}}{p_{i}DI_{i.pure}+p_{j}DI_{j.pure}}\;$$

Where NERDI (ij) is the net effect ratio of disease of the mixture of varieties i and j. DI_observed_ is the observed disease incidence or disease severity in the mixtures and DI_expected_ the expected disease incidence or disease severity based in the infections levels in pure stands. p_i_ and p_j_ are the proportion of the variety i and j in the mixture, respectively. DI_mix(i)_ is the disease incidence or the disease severity of the variety i in mixed plots and D_pure(i)_ the average disease infections in pure stands per block for variety i and likewise for the variety j. A NERDI value less than 1 indicates a higher resistance performance in the mixture than in the pure stand. Due to the low disease pressure during the experiment, only one variety (DOURADO PRECOCE) developed symptoms on leaves. As a result, the NERDI indicator was calculated for half of the mixtures, i.e. those that included this susceptible variety. Then, *Pyricularia oryzea* developed symptoms on panicles in two varieties: DOURADO PRECOCE and EARLY MUTANT IAC 165, but only at the IVO site. The NERDI on panicle was calculated in plots including these two varieties.

### 2.7 Statistical analysis

All the statistical analyses were done using R version 4.2.2 (2022-10-31 ucrt).We assessed the effects of the different modalities (pure stand and mixtures) and their interactions with the site on grain and biomass yields, diseases resistance and striga infestation using a mixed linear model (function lmer package lme4) (Banta et al., 2010; Bates et al., 2015). Association types, including cultural condition, different arrangement, and the main plot, were considered as factors with fixed effects. The block and plot positions within each site were included as random factors. Adjusted means were calculated using the lsmean function from the emmeans package (Searle, 2012), wich takes into account the variability introduced by the random factors. Mean comparisons were performed using the cld function from the multcomp package (Dunnett, 1955; Hothorn et al., 2008). This function assigns letters to the different levels of the modalities being compared (i.e., ‘Culture condition (pure stand vs mixture)’, ‘Site’, and ‘Arrangement’) based on their statistical differences.

The differences between RYT and NERDI and the value 1 (the reference value without any mixture effect) were assessed using the one-sample Student’s t-test. We assessed a linear model considering association type (i.e., ‘Culture condition (pure stand vs mixture)’, ‘spatial arrangement’, ‘main plot’) and site as fixed effects and the block and plot positions within each site were added as random factors. Adjusted means of each index were again calculated by lsmean. Then we compared the means between sites and the means for modalities of mixture repartition. Taking into account the comparison between modalities in each site and in general, the most efficient spatial arrangements was selected. Then we detailed the component mixtures of varieties in the identified spatial arrangement to highlight the one(s) inducing best performances. In this more efficient spatial arrangement (L1/3), the characteristics of each variety were compared across various proportions, both in pure stands and mixtures. Comparisons were performed using a Fisher’s multiple comparison test following an ANOVA, treating each trait as the dependent variable and the proportions as the independent variable for each variety.

## 3 Results

### 3.1 Effect of varietal mixtures on yield and resistance to biotic constraints

The results show that the variety EARLY MUTANT IAC 165 was the most productive in terms of grain yield (p<0.001) and it also performed well in biomass yield even the p-value was not significant between the monocultures of the four variety (**Table 2**). Mixtures, on the other hand, had a significant positive impact on both grain and biomass yields (p< 0.001) (**Table 2**). On average, varietal mixtures improved grain yield by 265g.m^-^² and biomass yield by 236 g.m^-^². These positive effects of mixtures were observed in both sites, although there were significant differences in yields between the two sites. Specifically, site IVO, with high fertilizer inputs, had significantly higher yields of 631g.m^-^² for grain and 363g.m^-^² for biomass, compared to the ANK site. The interactions between crop conditions and sites also had significant effects (p<0.05) (**Table 2**). *Pyricularia oryzea* and BLB have significantly negative association with grain yield (p<0.001) (**Figure S1**).

**Table 2:** Analysis of the effect of cropping system and the site experimental condition on upland rice production using ANOVA: means and standard errors for grain yield and biomass averaged over varietal mixtures pure stands and each site.

	Grain yield (g.m²)				Biomass yield (g.m²)			
*Crop conditon*								
Pure stand	747	±	34	***b***	948	±	50	***b***
Mixtures	1012	±	32	***a***	1184	±	44	***a***
***p-value***	***<0.001***		***		***<0.001***		***	
*Sites*								
Site ANK	596	±	44	***b***	892	±	62	***b***
Site IVO	1227	±	44	***a***	1254	±	62	***a***
***p-value***	***<0.001***		***		***0.002***		**	
Crop condition * Site								
***p-value***	***<0.05***		**		***0.1***		***NS***	
*Monocultures*								
EARLY MUTANT IAC 165 (A)	897	±	40	***a***	1052	±	66	***a***
FOFIFA 152 (B)	713	±	30	***bc***	823	±	34	***a***
FOFIFA 154 (C)	642	±	35	***c***	1004	±	33	***a***
DOURADO PRECOCE (D)	735	±	28	***b***	911	±	44	***a***
***p-value***	***<0.001***		***		***0.1***		***NS***	

*, **, *** p-value indicating significant differences at the 5%, 1%, and 0.1% levels of confidence, respectively. The letters ‘a’ and ‘b’ indicate significant differences at the 5% level of confidence, with ‘a’ denoting the higher mean value and ‘b’ the lower mean value. Levels with the same letter are not significantly different, while levels with different letters are considered significantly different from each other. NS, non-significant.

The values in bold represent the p-values, while the letters denote the multiple comparison groups resulting from the Fisher's test.

Regarding disease impact, mixtures showed significantly lower *Pyricularia oryzea* infection levels on leaves and panicles compared to pure stands (**Table 3**). Incidence on leaves was reduced by 5% (41% in mixtures versus 46% in pure stands), and the average number of lesions per leaf was reduced by three in all mixtures (5 lesions per hill in mixtures versus 8 lesions in pure stands). *Pyricularia oryzea* incidence on panicles decreased by half in mixtures, resulting in a 9 lesions reduction in the average number of lesions on panicles. However, no significant differences were observed for BLB and striga infestation rates (**Table 3**).

**Table 3:** Analysis of the effects of cropping and experimental conditions on biotic stresses using ANOVA: means and standard errors of disease incidence and severity for ***Pyriularia oryzea***, BLB, and Striga infestation averaged over varietal mixtures and pure stands and each site.

	Pyricularia on leaf incidence (%)				Pyricularia on leaf severity (nb)				Pyricularia on panicle incidence (%)				Pyricularia on panicle severity (nb)				BLB (%)				Striga infection (nb)			
Crop conditon																								
Pure stand	46	±	2	***a***	8	±	1	***a***	12	±	0.11	***a***	21	±	3	***a***	20	±	2	***a***	70	±	15	***a***
Mixtures	41	±	1	***b***	5	±	1	***b***	6	±	0.06	***b***	12	±	1	***b***	20	±	2	***a***	66	±	15	***a***
***p-value***	***0.02***		*		***<0.001***		***		***<0.001***		***		***0.01***		**		***0.9***		***NS***		***0.3***	***NS***		
Site																								
Site ANK	45	±	2	a	10	±	1	a		0				0				0			70	±	6	
Site IVO	42	±	2	b	3	±	1	b	7.5	±	0.1		14	±	2		20	±	2		0			
***p-value***	***0.02***		*		***<0.001***		***																	
Crop condition * Site																								
***p-value***	***0.11***		***NS***		***<0.001***		***		***NA***				***NA***				***NA***				***NA***			
Monocultures																								
EARLY_MUTANT_IAC_165		0				0			8.3	±	0.1	***b***	12	±	4	***b***	9	±	2	***c***	82	±	15	***a***
FOFIFA 152		0				0				0				0			23	±	11	***b***	73	±	12.5	***a***
FOFIFA 154		0				0				0				0			31	±	15	***a***	77	±	14.5	***a***
DOURADO_PRECOCE	46	±	2		8	±	1		16	±	0.1	***a***	30	±	4	***a***	17	±	14	***bc***	49	±	13.3	***b***
***p-value***									***0.02***		*						***<0.001***		***		***<0.01***		**	

*, **, *** p-value indicating significant differences at the 5%, 1%, and 0.1% levels of confidence, respectively. The letters ‘a’ and ‘b’ indicate significant differences at the 5% level of confidence, with ‘a’ denoting the higher mean value and ‘b’ the lower mean value. Levels with the same letter are not significantly different, while levels with different letters are considered significantly different from each other. NS, non-significant. NA, non-existent value.

The values in bold represent the p-values, while the letters denote the multiple comparison groups resulting from the Fisher's test.

The results of the site-specific analysis reveal significant differences between the two experimental sites. The IVO site showed significantly lower values of *Pyricularia oryzea* incidence and severity on leaves compared to the ANK site. Specifically, the differences between the two sites were 3% in *Pyricularia oryzea* incidence (p = 0.02) and a reduction of 7 lesions number in *Pyricularia oryzea* severity (p<0.001). Interestingly, the pathogen *Pyricularia oryzea* only affected panicles at the IVO site, with an incidence rate of 6% and an average of 9 lesions in severity. Additionally, Bacterial Leaf Blight (BLB) infection was observed only at the IVO site, while striga infestation occurred solely in the experiment at the ANK site. In summary, the highly fertilized IVO site demonstrated higher yields and lower *Pyricularia oryzea* infection levels compared to the ANK site.

### 3.2 Relative productivity and disease resistance of mixture

**Figure 2:**
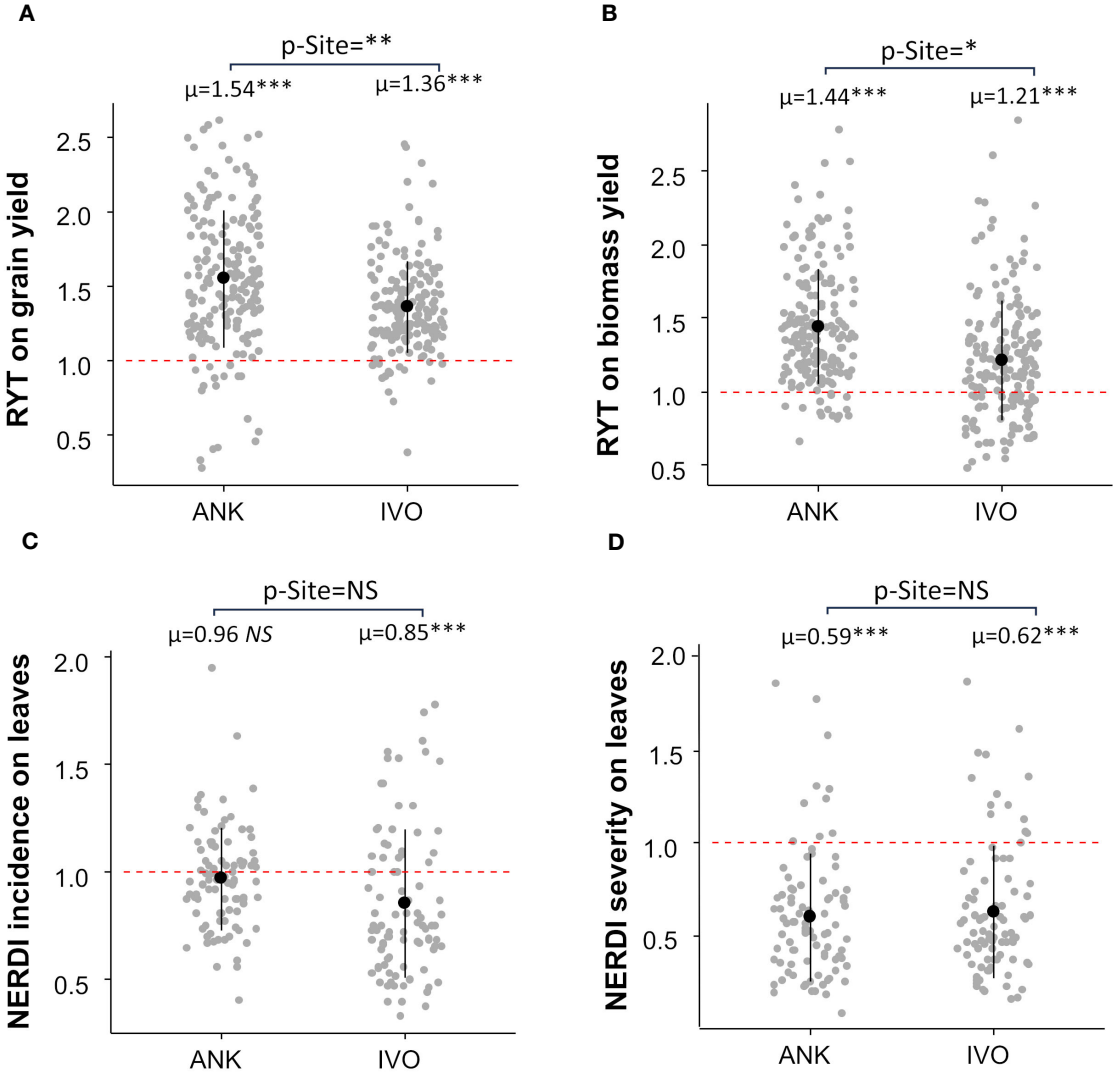
Relative Yield Total (RYT) of grain **(A)** and biomass yield **(B)** and Net Effect Ratio of Disease (NERDI) of varietal mixtures in two sites. **(C)** showed NERDI incidence on leaves and **(D)** showed NERDI severity on leaves. Each site average was compared with a reference value of 1 by a t-test; p-values are indicated in the top of each plot as “p-Site”. RYT and NERDI averages per site are shown i.e., µ=1.54 above scatterplots. The site effect was assessed by linear model analysis and p-values are indicated by stars next to averages, p-value < 0.05 = *; p-value < 0.01= **; p-value < 0.001= ***. NS, non-significant.

The RYT analysis demonstrated an overall 45% increase in grain yield and a 33% increase in biomass yield in mixtures compared to monocultures (**Figure 2**). The effect of mixtures was consistent across both sites for grain yield, with a gain of 54% in the ANK site and 36% in the IVO site, respectively (**Figure 2A**). however, there was a large difference of 22% in biomass yield between the two sites. The biomass yield gain through mixtures in the ANK site was the double of the biomass gain in IVO site (**Figure 2B**).

Results also demonstrated an overall reduction in *Pyricularia oryzea* infection on leaves and panicles in mixtures. In comparison to pure-stands, mixtures showed an average 10% decrease in disease incidence on leaves (**Figure 2C**) and a 39% decrease in disease severity on leaves (**Figure 2D**). The resistance to *Pyricularia oryzea* on leaves was very effective at the IVO site, with disease incidence reduced by 15% and severity by 38% (**Figures 2C, D**). However, this was not the case at the ANK site, where the incidence of *Pyricularia oryzea* on leaves was not reduced by the mixture (**Figure 2C**). Nevertheless, for this site, we observed a reduction of 41% in disease severity (**Figure 2D**). The *Pyricularia oryzea* infection was reduced by 49% in both NERDI incidence and severity on the panicle at the IVO site since there was no panicle infection in the ANK site (mean NERDI = 0.51 p-value<0.001 for both incidence and severity).

**Figure 3:**
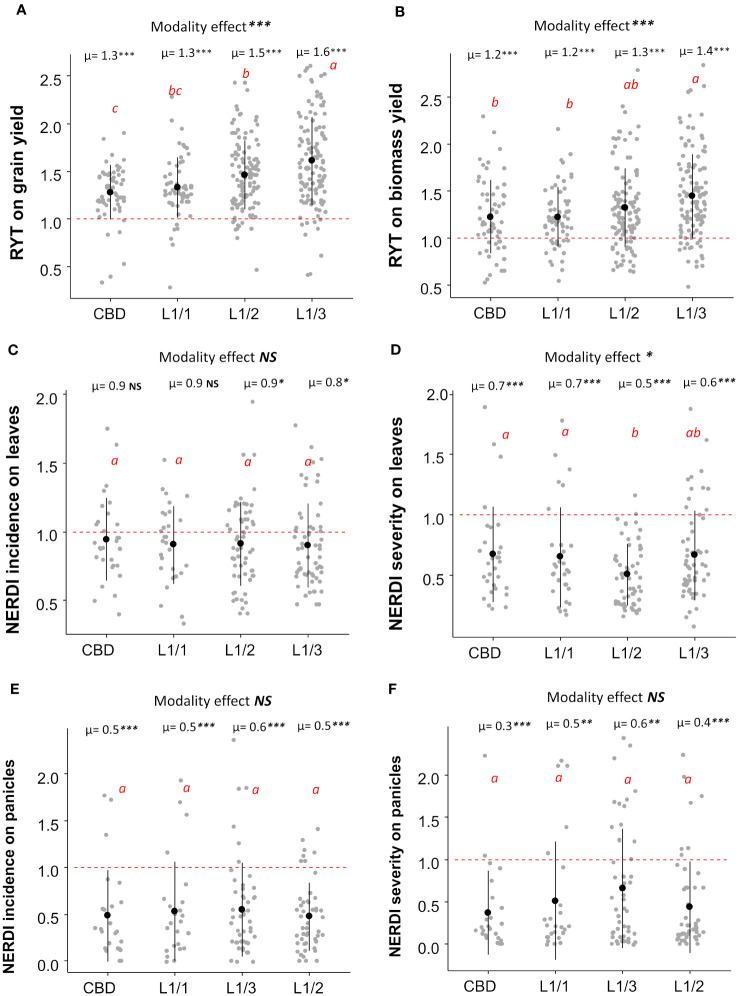
Average RYT for grain **(A)** and biomass yield **(B)** and NERDI values for disease incidence **(C)** and severity **(D)** on leaves with NERDI incidence on panicles **(E)** and NERDI severity on panicles **(F)** of different spatial arrangements of rice mixtures. The modality effect was assessed by ANOVA, and the p-value as indicated by stars in the top of each panel as “Modality effect ***”. The letters 'a', ‘ab’,'b', ‘bc’ and ‘c’ indicate significant differences between arrangements at the 5% level of confidence. ‘a’ indicates the higher value and ‘c’ the lower value. Each modality average (i.e., µ=1.3) was compared with a reference value of 1 by a t-test; p-values are indicated in the top of each plot, near the averages (i.e., µ=1.3***). p-value < 0.05 = *; p-value < 0.01= **; p-value < 0.001= ***). NS, non-significant.

Regarding the spatial arrangement, there were no significant differences between lines and checkerboard patterns within the L1/1 arrangement for all the considered, variables including yields and disease resistance (**Figure 3**). The most unbalanced variety repartition (L1/3) induced the highest RYT for grain yield (mean =1.60) in both sites (**Figure S2**), while L1/1 and L1/2 did not differ significantly (**Figure 3A**). Similarly, the most unbalanced repartition (L1/3) was the most efficient for biomass yield (mean RYT= 1.40), with no significant differences observed for the other three repartitions (**Figure 3B**). All other repartitions had similar performances in reducing disease incidence on leaves, but the L1/3 repartition had the lowest NERDI value, which was significantly different for one (p<0.05) (**Figure 3C**). For the disease severity reduction on leaves, the L1/2 and L1/3 repartition were the most efficient with an average reduction of 50% and 40% respectively (**Figure 3D**). At the panicle level, the effect of different spatial arrangement on both NERDI incidence and severity was consistently positive with a reduction of the disease impact, but there were no significant differences among the modalities (**Figures 3E, F**). Site-specific results for the effects of different arrangements are available in the Supplementary Information in **Figure S2**.

**Figure 4:**
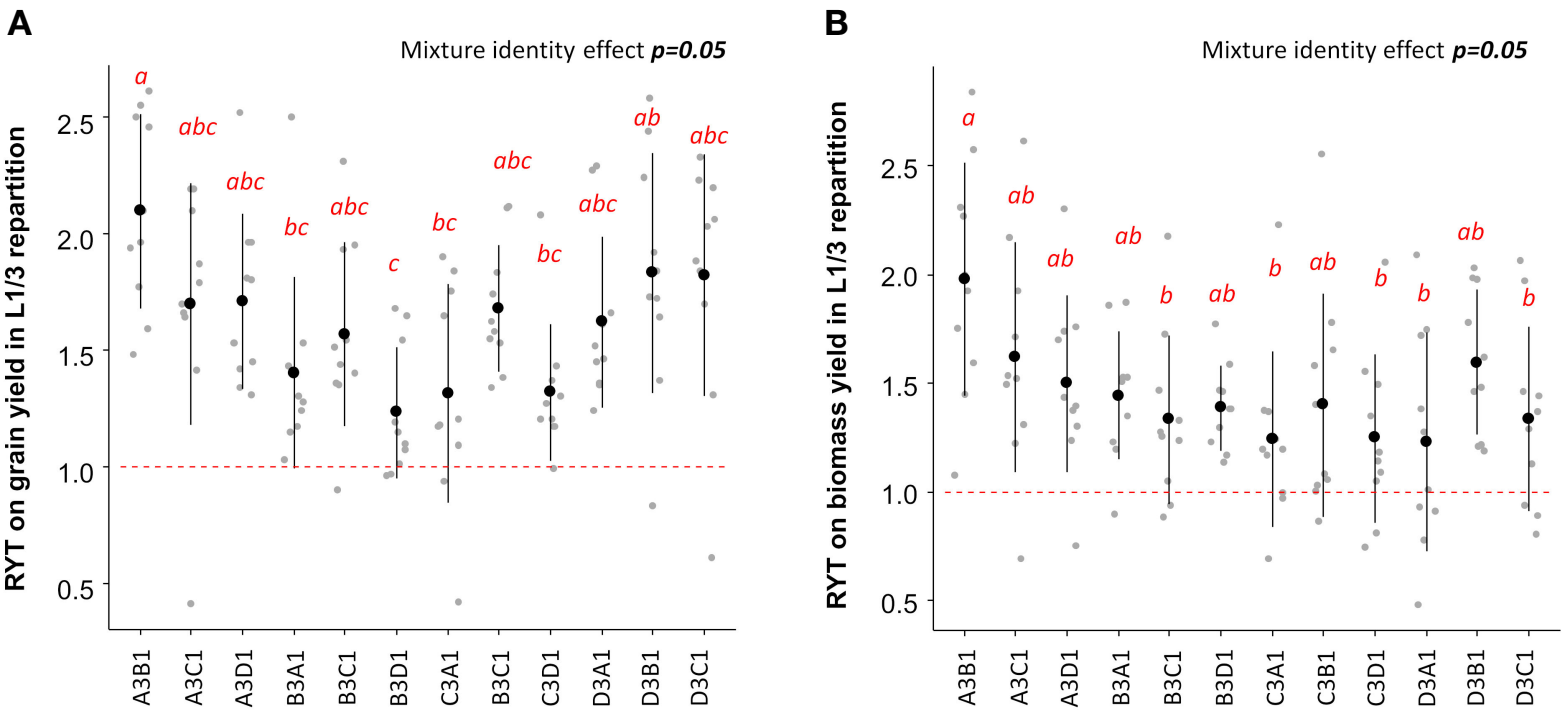
RYT values for grain **(A)** and biomass **(B)** yield for L1/3 repartition. Mixture effects are assessed using ANOVA indicated in the right top of each panel. Letters ‘a’ to ‘c’ indicate significant differences at the 5% level of confidence with ‘a’ indicating a higher value and ‘c’ the lower value. The first letter in the mixture name, i.e A for the association A3B1, indicate the variety that represent 75% of hills within the mixtures and the second letter indicate the variety that represent 25% of the mixture. A stands for EARLY MUTANT IAC 25; B stands for FOFIFA 152; C stands for FOFIFA 154;and D stands for DOURADO PRECOCE.

Given that the L1/3 repartition was the most efficient for increasing grain and biomass yield, it was chosen to identify the best performing varietal mixtures based on RYT. The highest RYT values for grain and biomass yield were observed when the variety EARLY MUTANT IAC 165 represented 75% of the plot (**Figures 4A, B**). According to **Table 2**, EARLY MUTANT IAC 165 was the most productive variety in grain and biomass yields. Its positive impact in mixtures may be attributed to the fact that the hills of this variety produced more tillers and leaves in mixtures than in pure stands, possibly due to an alleviation of the intra-varietal competition. This effect was particularly pronounced when EARLY MUTANT IAC 165 was associated with the varieties FOFIFA 152 and FOFIFA 154. Notably, these two varieties are resistant to *Pyricularia oryzea*, and were not affected by this pathogen in our study. On the other hand, the association composed by 25% EARLY MUTANT IAC 165 and 75% of DOURADO PRECOCE had the lowest RYT for biomass (**Figure 4B**). In this case the association of two sensitive varieties to *Pyricularia oryzea* attack (**Table 3**) with 75% of plants coming from the most sensitive variety seemed to counterbalance the positive effect of EARLY MUTANT IAC 165 on mixture performances (**Figure 4**). Similar trends were observed at the site-specific level (**Figure S3**).

## 4 Discussion

This study demonstrated that binary varietal upland rice mixtures had significantly higher grain and biomass yield compared to the varieties in pure stand, indicating the benefits of varietal mixtures. The Relative Yield Total (RYT) indicated an average of 45% gain in grain yield and 33% gain on biomass yield when applying varietal combination. These benefits of intercropping can be interpreted as improved land use efficiency (Mead and Willey, 1980; Werf, 2021).This finding is consistent with and confirms previous studies on varietal mixtures of rice or other crops (Borg et al., 2018; Montazeaud et al., 2020). The study also demonstrated lower *Pyricularia oryzea* infection on both leaves and panicles in mixtures, highlighting that varietal mixtures can effectively reduce impact of this fungal disease on crop development as previously demonstrated by Gallet et al. (2014) and Raboin et al. (2012). This reduction is an epidemiological phenomenon when mixtures were composed of varieties with varying levels of resistance. It was particularly evident in the susceptible variety (Raboin et al., 2012; Zhu et al., 2000). The same fact was observed in our study since the *Pyricularia oryzea* only infected the plot where the most susceptible variety (DOURADO PRECOCE) was present. Disease incidence and severity were higher in pure stands of this variety compared to mixtures. However, no significant differences between mixtures and pure stands were found for Bacterial Leaf Blight (BLB) and striga infestation.

Significant variations in yields were observed between the experimental sites according to the **Table 2**. The IVO site, which was highly fertilized, had significantly higher yields compared to the ANK site. These site differences induce variations in mixture effects, particularly for biomass production, with a stronger positive effect of the mixture in the low fertility, ANK site (**Figure 2**). The same trend was observed for grain yield, though the differences were not statistically significant. According to these findings, the varietal mixtures were more pronounced in less fertilized field conditions. These results are in line with the literature that demonstrate that environmental factors and nutrients availability can affect the mechanisms involved in the establishment of the positive effect of varietal mixture (Barot et al., 2017; Grzyb et al., 2020). Moreover, they are consistent with the stress gradient hypothesis, which suggests predictable variation in net plant-plant interactions based on environmental context, with more positive effects of these interactions in the case of low level of resources availability (Brooker et al., 2014). These findings highlight the importance of tailoring intercropping strategies to local environmental conditions for optimal crop performance.

It was demonstrated that soil organic matter content, which is a major driver of soil fertility, could impact mixture performance, i.e. the more organic matter available, the lower the mixture effect and thus the relevance of using varietal mixtures (Reiss and Drinkwater, 2018). This stands in contrast to our findings, and we can hypothesise that this is due to the fact that the fertility of the low soil organic matter content site (Ivory) was improved through fertilisation. This confirms that positive effects of varietal mixtures were stronger under low fertility conditions, as stated by (Reiss and Drinkwater, 2018) and tends to demonstrate that in our case fertilizer input was more important to determine the mixture effect than soil organic matter content. Varietal mixtures can be beneficial in low-fertility soils due to the distinct resource acquisition patterns of different plant varieties (Lambers et al., 2008) and their potential to capture solar radiation differently (Richards, 2000). These varietal differences may lead to compatibility among varieties, facilitating efficient resource utilization (Li et al., 2018). Moreover, our findings supported previous results of Kiær et al. (2012) showing that within field intraspecific diversity allow crops to better respond to environmental factors and especially nutrient availability (Baraibar et al., 2020). However, this assumption needs to be qualified with the discovery by Dahlin et al. (2020), which suggests that the resource niches achieved depend on the genotypes’ identity in the mixture, and this functional mechanism might be influenced by the trait plasticity of individual genotypes leading to different phenotypes. The reasons for improved performance of mixtures is largely to be explored.

Grime (1977) pointed out that in a less fertile site, negative interactions among plants are limited due to an alleviation of the competition from large size individuals when resource availability is restricted. Another reason could be that in low fertility conditions, differences in resources acquisition strategies among genotypes should be more important for the plant community functioning. This is due to a scarcity of resources, thus generating a strong positive effect of plant complementarity on the performance of the mixture (Cook-Patton et al., 2011). These combined hypotheses may explain the positive impacts of inter and intraspecific diversity on use of community-level resource-such as light, water, and nutrients; resulting in improved overall resource utilization and increased crop productivity (Bakker et al., 2016; Loreau, 1998; Craven et al., 2016; Montazeaud et al., 2018). In contrast, we can hypothesise that the benefits of mixing in a more fertilized site were not as pronounced due to a dominance (Grime, 1977) effect of the most productive varieties that perform well in both pure stand and monoculture, limiting the relevance of the mixture. This may also be due to the fact that the effect of differential root exploitation is no longer emphasised in the complementarity effect when resources are not constrained (Elser et al., 2014).

In our study, varietal mixtures demonstrated efficiency to reduce *Pyricularia oryzea* incidence and severity. These findings are in line with previous research that has demonstrated the potential of mixtures to reduce pest and disease pressure through diversity-mediated pest suppression via specific ecological processes (Pélissier et al., 2021). These results can be related to several ecological mechanisms: the lower *Pyricularia oryzea* infection could be attributed to reduced host-plant density and increased diversity, making mixtures less favourable to the spread of pathogens (Ekroth et al., 2019). This mechanism is described in ecological studies as the dilution effect or the barrier effect, whereby the introduction of non-susceptible species or genotypes can limit the spread of the disease due to a physical barrier effect, (Li et al., 2007; Zhu and Morel, 2019). However, we did not detect any positive effect of the varietal mixtures on striga infestation, which is contradictory to previous research demonstrating that genetically homogenous populations are more susceptible to parasites and pathogens (Ekroth et al., 2019). Studies conducted on millet varietal mixtures in Senegal have similar results to our findings, showing a neutral effect of mixtures on striga and weed infestation (Cissé et al., 2023). Overall, more research is needed to fully understand the relationship between pure stand and the spread of BLB and striga infestation (**Table 3**). Furthermore, it is important to consider local factors such as environmental conditions and rice variety choice that can influence the specific results.

Regarding the spatial arrangement, the most unbalanced repartition showed a higher RYT for grain yield in both sites, highlighting that the balance between the varieties is not a prerequisite to obtaining a positive effect of mixtures (Newton and Skelsey, 2023). These results confirm findings of Zhang et al. (2011), who found a variation of biomass yield in different arrangements of wheat-soybean intercropping, thus emphasizing the benefits of an unbalanced arrangement for grain yield. Those findings reveal that the performance of varietal mixtures in agroecosystems is a complex interplay between different ecological mechanisms. Contrary to a strong complementarity effect that could alleviate resource competition, research like Cardinale et al. (2007) highlights that the success of these mixtures often hinges on a selection effect. This effect is characterized by the dominance of a particular component within the mixture, driving its overall performance. This suggests that the presence of a highly successful individual variety can play a pivotal role in determining the overall success of the mixture (Loreau and Hector, 2001; Tilman et al., 2006). These insights underlined the intricate dynamics that dictate the outcomes of varietal mixtures in agriculture, where the dominance of certain components can outweigh the potential benefits of resource complementarity (Lehman and Tilman, 2000).This hypothesis appears to be confirmed by our result because of the dominant genotype in the mixture with the highest RYT, i.e. the EARLY MUTANT IAC 165. This variety produced more leaves biomass and had taller plants than the other varieties, two characteristics which are indicative of high competitive ability (Grime, 1977). Further substantiating these findings, **Table 4** presented complete yield data for varieties in both pure stand and in L1/3 repartitions as 75% or 25% proportions. Observed characteristics of the varieties under these repartitions are also available in this table. Additionally, we may associate this highly competitive and productive variety with smaller varieties displaying high root biomass (FOFIFA 152 and FOFIFA 154). This contributes to the improved yield performance of the mixture, leading us to suspect that spatial complementarity for light interception, as well as unbalanced competition for above- and below-ground resources, could explain the performance of the mixture. Our study highlights the fact that selecting specific varietal compositions and unbalanced spatial arrangements can improve grain and biomass yields in upland rice mixtures.

**Table 4:** Performance in production and characteristics of the varieties at different proportions (75% and 25%) in the L1/3 repartition compared to pure stands.

Variety name	Proportion	Grain yield (g.m²)				Biomass yield (g.m²)				Vegetative time (day)				Flowering time (day)				Plant heigth (cm)				Tiller number (nb)				Leaves number (nb)				Aerial biomass (g)			
EARLY MUTANT IAC_165 (A)	25%	254	±	10	***c***	911	±	29	***a***	85	±	3	***a***	103	±	9	***a***	113	±	6	***a***	13	±	5	***ab***	62	±	19	***ab***	278	±	13	***b***
	75%	1 089	±	45	***a***	407	±	21	***b***	87	±	7	***a***	106	±	8	***a***	107	±	18	***a***	13	±	5	***a***	66	±	20	***a***	403	±	24	***a***
	100%	897	±	40	***b***	1 035	±	66	***a***	85	±	3	***a***	105	±	7	***a***	106	±	21	***a***	11	±	4	***b***	51	±	16	***b***	370	±	18	***ab***
FOFIFA 152 (B)	25%	338	±	14	***b***	1 027	±	31	***a***	84	±	4	***ab***	102	±	10	***a***	101	±	21	***a***	14	±	5	***a***	63	±	22	***a***	326	±	19	***a***
	75%	733	±	29	***a***	320	±	11	***c***	85	±	4	***a***	101	±	10	***a***	98	±	21	***a***	14	±	5	***a***	60	±	20	***a***	315	±	17	***a***
	100%	713	±	28	***a***	862	±	33	***b***	84	±	4	***b***	100	±	11	***a***	99	±	20	***a***	15	±	5	***a***	66	±	19	***a***	303	±	16	***a***
FOFIFA 154 (C)	25%	332	±	16	***b***	960	±	28	***a***	86	±	3	***a***	104	±	9	***a***	98	±	19	***a***	13	±	5	***a***	63	±	25	***a***	326	±	17	***a***
	75%	701	±	30	***a***	286	±	7	***b***	85	±	4	***a***	107	±	13	***a***	101	±	22	***a***	14	±	4	***a***	59	±	19	***a***	282	±	16	***a***
	100%	642	±	30	***a***	995	±	44	***a***	85	±	3	***a***	105	±	11	***a***	106	±	60	***a***	13	±	5	***a***	61	±	25	***a***	307	±	17	***a***
DOURADO PRECOCE (D)	25%	257	±	11	***c***	898	±	19	***a***	85	±	3	***ab***	105	±	10	***a***	109	±	21	***ab***	12	±	4	***a***	57	±	18	***a***	342	±	18	***a***
	75%	946	±	45	***a***	305	±	8	***b***	86	±	4	***a***	104	±	9	***a***	100	±	20	***b***	12	±	4	***a***	56	±	18	***a***	255	±	15	***a***
	100%	735	±	35	***b***	898	±	34	***a***	84	±	3	***b***	104	±	14	***a***	111	±	19	***a***	11	±	3	***a***	54	±	15	***a***	440	±	17	***a***

Mixture values represent the mean performance of the specific variety when it is combined with the three other varieties.

The values in bold represent the p-values, while the letters denote the multiple comparison groups resulting from the Fisher's test.

The results of this study indicated important benefits of varietal mixtures, albeit these results had limitations, as they were specific to the experimental conditions and locations. Extrapolating the results to different environmental conditions or rice varieties should be treated with caution, as local factors such as soil type, climatic conditions, and genotype selection can influence outcomes. While the study demonstrated the effectiveness of mixtures in reducing *Pyricularia oryzea* infection, no significant differences were found for BLB and striga infestation. This suggests that the impact of mixtures on different diseases may vary. Further research is needed to fully understand the relationship between pure stands and the spread of these specific infections. The study, furthermore, provided insights into the positive effects of varietal mixtures on crop performance, however, the underlying mechanisms driving these outcomes remain largely unexplored. Future studies should aim to investigate the specific ecological processes and interactions contributing to the observed benefits. Our results highlight the potential benefits of an unbalanced spatial arrangement regarding grain yield. However, the specific reasons behind these findings, such as the selection effect, driven by the dominance of a particular genotype, require further investigation. Additionally, the relationship between spatial arrangement, complementarity effects, and resource competition must be explored further. Lastly, the experiment duration of a single growing season limited our insights into the sustainability and stability of the observed benefits. Long-term experimentation is needed to confirm the above results.

Our study highlighted the benefits of varietal mixtures in upland rice in the mid-west of Madagascar. The highland rice production systems of Madagascar often face challenges such as limited access to inputs such as fertilizers and pesticides. Intercropping can help to address some of these challenges by reducing the reliance on external inputs and enhancing ecosystem functioning. However, understanding ecological mechanisms that drive interaction among varieties is crucial for optimizing mixture composition and improving rice production sustainability. However, further research is needed to explore these mechanisms in more detail and to assess their long-term implications for rice production in the context of highland agroecosystems in Madagascar.

## Data availability statement

The data and the script for data analysis are available at the following link: https://figshare.com/articles/dataset/Assessing_the_impact_of_rice_varietal_mixtures_on_crop_performance_in_Madagascar/24488386.

## Author contributions

KR: Conceptualization, Data curation, Formal analysis, Investigation, Methodology, Software, Supervision, Writing – original draft, Writing – review & editing. BM: Conceptualization, Funding acquisition, Investigation, Project administration, Resources, Validation, Writing – review & editing. CV: Conceptualization, Data curation, Formal analysis, Funding acquisition, Resources, Writing – review & editing. KB: Investigation, Writing – original draft, Writing – review & editing. RP: Supervision, Validation, Writing – review & editing. JM: Investigation, Methodology, Validation, Writing – review & editing. EB: Methodology, Software, Writing – review & editing. FF: Conceptualization, Data curation, Formal analysis, Methodology, Supervision, Validation, Writing – original draft, Writing – review & editing.

## References

[B1] Altieri M. A. (1999). The ecological role of biodiversity in agroecosystems. Agriculture Ecosyst. Environ. 74 (19–31), 13. doi: 10.1016/B978-0-444-50019-9.50005-4

[B2] Bakker L. M. Mommer L. ruijven j. v. (2016). Can root trait diversity explain complementarity effects in a grassland biodiversity experiment? J. Plant Ecol. 11 (1), 73–84. doi: 10.1093/jpe/rtw111

[B3] Balasubramanian V. Sie M. Hijmans R. J. Otsuka K. (2007). “Increasing Rice Production in Sub-Saharan Africa: Challenges and Opportunities,” in Advances in Agronomy, vol. 94. (Elsevier), 55–133. doi: 10.1016/S0065-2113(06)94002-4

[B4] Banta J. A. Stevens M. H.H. Pigliucci M. (2010). A comprehensive test of the ‘Limiting resources’ Framework applied to plant tolerance to apical meristem damage. Oikos 119 (2), 359–695. doi: 10.1111/j.1600-0706.2009.17726.x

[B5] Baraibar B. Murrell E. G. Bradley B. A. Barbercheck M. E. Mortensen D. A. Kaye J. P.. (2020). Cover crop mixture expression is influenced by nitrogen availability and growing degree days. PLOS ONE 15 (7), e0235868. doi: 10.1371/journal.pone.0235868 32716963 PMC7384630

[B6] Barot Sébastien Allard V. Cantarel Amélie Enjalbert Jérôme Gauffreteau A. Goldringer I.. (2017). Designing mixtures of varieties for multifunctional agriculture with the help of ecology. A review. Agron. Sustain. Dev. 37 (2), 135. doi: 10.1007/s13593-017-0418-x

[B7] Bates D. Mächler M. Bolker B. Walker S. (2015). Fitting linear mixed-effects models using lme4. J. Stat. Software 67 (October), 1–48. doi: 10.18637/jss.v067.i01

[B8] Boero F. (2015). From darwin’s origin of species toward a theory of natural history. F1000Prime Rep. 7 (May), 1–13. doi: 10.12703/P7-49 PMC4447030 26097722

[B9] Borg J. Kiær L. P. Lecarpentier C. Goldringer I. Gauffreteau A. Saint-Jean S.. (2018). Unfolding the potential of wheat cultivar mixtures: A meta-analysis perspective and identification of knowledge gaps. Field Crops Res. 221 (May), 298–313. doi: 10.1016/j.fcr.2017.09.006

[B10] Breumier P. Ramarosandratana A. Ramanantsoanirina A. Brocke K. v. Marquié C. Dabat Marie-Hélène. (2018). Évaluation participative des impacts de la recherche sur le riz pluvial d’altitude à Madagascar de 1980 à 2015. Cahiers Agricultures 27 (1), 150045. doi: 10.1051/cagri/2017065

[B11] Brooker R. W. Bennett A. E. Cong W.-F. Daniell T. J. George T. S. Hallett P. D.. (2014). Improving intercropping: A synthesis of research in agronomy, plant physiology and ecology. New Phytol. 206 (1), 107–117. doi: 10.1111/nph.13132 25866856

[B12] Cardinale B. J. Duffy J.E. Gonzalez A. Hooper D. U. Perrings C. Venail P.. (2012). Biodiversity loss and its impact on humanity. Nature 486 (7401), 59–67. doi: 10.1038/nature11148 22678280

[B13] Cardinale B. J. Matulich K. L. Hooper D. U. Byrnes J. E. Duffy E. Gamfeldt L.. (2011). The functional role of producer diversity in ecosystems. Am. J. Bot. 98 (3), 572–925. doi: 10.3732/ajb.1000364 21613148

[B14] Cardinale B. J. Palmer M. A. Collins S. L. (2002). Species diversity enhances ecosystem functioning through interspeci®c facilitation. Nautre 415, 4. doi: 10.1038/415426a 11807553

[B15] Cardinale B. J. Wright J. P. Cadotte M. W. Carroll I. T. Hector A. Srivastava D. S.. (2007). Impacts of plant diversity on biomass production increase through time because of species complementarity. Proc. Natl. Acad. Sci. 104 (46), 18123–18285. doi: 10.1073/pnas.0709069104 17991772 PMC2084307

[B16] Cissé A. Clermont-Dauphin C. Sall SaïdouN. Gie S. Groupement M. P. Ndiaye A.. (2023). Sahelian smallholders’ Varietal mixtures reconcile yield and agrobiodiversity conservation. Basic Appl. Ecol. 67 (March), 48–60. doi: 10.1016/j.baae.2022.12.006

[B17] Cook-Patton S. C. McArt S. H. Parachnowitsch A. L. Thaler J. S. Agrawal A. A. (2011). A direct comparison of the consequences of plant genotypic and species diversity on communities and ecosystem function. Ecology 92 (4), 915–235. doi: 10.1890/10-0999.1 21661554

[B18] Craven D. Isbell F. Manning P. Connolly J. Bruelheide H. Ebeling A.. (2016). Plant diversity effects on grassland productivity are robust to both nutrient enrichment and drought. Philos. Trans. R. Soc. B: Biol. Sci. 371 (1694), 20150277. doi: 10.1098/rstb.2015.0277 PMC4843698 27114579

[B19] Creissen H. E. Jorgensen T. H. Brown J. K. M. (2016). Increased yield stability of field-grown winter barley (Hordeum vulgare L.) varietal mixtures through ecological processes. Crop Prot. 85 (July), 1–8. doi: 10.1016/j.cropro.2016.03.001 27375312 PMC4862440

[B20] Dahlin I. Kiær L. P. Bergkvist Göran Weih M. Ninkovic V. (2020). Plasticity of barley in response to plant neighbors in cultivar mixtures. Plant Soil 447 (1–2), 537–551. doi: 10.1007/s11104-019-04406-1

[B21] De Wit C. T. (1960). On competition. Verslagen Landbouwkundige Onderzoekigen 66, 1–82.

[B22] Dunnett C. W. (1955). A multiple comparison procedure for comparing several treatments with a control. J. Am. Stat. Assoc. 50, 1096–1121. doi: 10.1080/01621459.1955.10501294?journalCode=uasa20

[B23] Ekroth A. K. E. Rafaluk-Mohr C. King K. C. (2019). Diversity and disease: evidence for the monoculture effect beyond agricultural systems. Preprint. Ecol. 1–30. doi: 10.1101/668228

[B24] Elser J. J. Elser T. J. Carpenter S. R. Brock W. A. (2014). Regime shift in fertilizer commodities indicates more turbulence ahead for food security. PloS One 9 (5), e939985. doi: 10.1371/journal.pone.0093998 PMC4006770 24787624

[B25] FAO (2014). World Reference Base for Soil Resources 2014: International Soil Classification System for Naming Soils and Creating Legends for Soil Maps (Rome: FAO).

[B26] Feledyn-Szewczyk B. Kus J. Stalenga J. Berbeć A. K. Radzikowski P. (2016). “The Role of Biological Diversity in Agroecosystems and Organic Farming,” in Organic Farming - A Promising Way of Food Production. Ed. Konvalina P. (InTech). doi: 10.5772/61353

[B27] Fréville Hélène Montazeaud G. Forst E. David J. Papa R. Tenaillon M. I. (2022). Shift in beneficial interactions during crop evolution. Evolutionary Appl. 15 (6), 905–185. doi: 10.1111/eva.13390 PMC9234679 35782010

[B28] Gallet R. Bonnot François Milazzo Joëlle Tertois C. Adreit H. Ravigné V.. (2014). The variety mixture strategy assessed in a G × G experiment with rice and the blast fungus magnaporthe oryzae. Front. Genet. 4. doi: 10.3389/fgene.2013.00312 PMC3893683 24474958

[B29] Gaudio Noémie Violle C. Gendre X. Fort F. Mahmoud Rémi Pelzer E.. (2021). Interspecific interactions regulate plant reproductive allometry in cereal–legume intercropping systems. J. Appl. Ecol. 58 (11), 2579–2589. doi: 10.1111/1365-2664.13979

[B30] Gérardeaux E. Falconnier G. Gozé E. DeFrance D. Kouakou P.-M. Loison R.. (2021). Adapting rainfed rice to climate change: A case study in Senegal. Agron. Sustain. Dev. 41 (4), 575. doi: 10.1007/s13593-021-00710-2

[B31] Gerardeaux E. Giner M. Ramanantsoanirina A. Dusserre J. (2012). Positive effects of climate change on rice in Madagascar. Agron. Sustain. Dev. 32 (3), 619–275. doi: 10.1007/s13593-011-0049-6

[B32] Grime J. P. (1977). Evidence for the existence of three primary strategies in plants and its relevance to ecological and evolutionary theory. Am. Nat. 111 (982), 1169–1194. doi: 10.1086/283244

[B33] Grzyb A. Wolna-Maruwka A. Niewiadomska A. (2020). Environmental factors affecting the mineralization of crop residues. Agronomy 10 (12), 19515. doi: 10.3390/agronomy10121951

[B34] Hooper F. S. Chapin Ewel J. J. Hector A. Inchausti P. Lavorel S.. (2005). Effects of biodiversity on ecosystem functioning: A consensus of current knowledge. Ecol. Monogr. 75 (1), 3–35. doi: 10.1890/04-0922

[B35] Hooper D. U. Dukes J. S. (2003). Overyielding among Plant Functional Groups in a Long-Term Experiment: Overyielding among Plant Functional Groups. Ecol. Lett. 7 (2), 95–1055. doi: 10.1046/j.1461-0248.2003.00555.x

[B36] Hothorn T. Bretz F. Westfall P. (2008). Simultaneous inference in general parametric models - hothorn. Biometrical J. 50 (3), 2–18. doi: 10.1002/bimj.200810425 18481363

[B37] Jucker T. Coomes D. A. (2012). Comment on Plant species richness and ecosystem multifunctionality in global drylands. Science 337 (6091), 155–155. doi: 10.1126/science.1220473 22798584

[B38] Kiær L. P. Skovgaard I. Østergård H. (2012). Effects of inter-varietal diversity, biotic stresses and environmental productivity on grain yield of spring barley variety mixtures. Euphytica 185 (1), 123–385. doi: 10.1007/s10681-012-0640-1

[B39] Lambers H. Raven J. Shaver G. Smith S. (2008). Plant nutrient-acquisition strategies change with soil age. Trends Ecol. Evol. 23 (2), 95–103. doi: 10.1016/j.tree.2007.10.008 18191280

[B40] Lehman C. L. Tilman D. (2000). Biodiversity, stability, and productivity in competitive communities. Am. Nat. 156 (5), 195. doi: 10.1086/303402 29587515

[B41] Li C. Hoffland E. Kuyper T. W. Yu Y. Zhang C. Li H.. (2020). Syndromes of production in intercropping impact yield gains. Nat. Plants 6 (6), 653–605. doi: 10.1038/s41477-020-0680-9 32483328

[B42] Li L. Li S.-M. Sun J.-H. Zhou L.-L. Bao X.-G. Zhang H.-G.. (2007). Diversity enhances agricultural productivity via rhizosphere phosphorus facilitation on phosphorus-deficient soils. Proc. Natl. Acad. Sci. 104 (27), 11192–11965. doi: 10.1073/pnas.0704591104 17592130 PMC1899187

[B43] Li C. Stomph T.-J. Makowski D. Li H. Zhang C. Zhang F.. (2023). The productive performance of intercropping. Proc. Natl. Acad. Sci. 120 (2), e22018861205. doi: 10.1073/pnas.2201886120 PMC9926256 36595678

[B44] Li X.-F. Wang C.-B. Zhang W.-P. Wang L.-H. Tian X.-L. Yang S.-C.. (2018). The role of complementarity and selection effects in P acquisition of intercropping systems. Plant Soil 422 (1–2), 479–493. doi: 10.1007/s11104-017-3487-3

[B45] Loreau M. (1998). Biodiversity and ecosystem functioning: A mechanistic model. Proc. Natl. Acad. Sci. 95 (10), 5632–5636. doi: 10.1073/pnas.95.10.5632 9576935 PMC20430

[B46] Loreau Hector A. (2001). Partitioning selection and complementarity in biodiversity experiments. Nature 412 (6842), 72–76. doi: 10.1038/35083573 11452308

[B47] Malézieux E. (2012). Designing cropping systems from nature. Agron. Sustain. Dev. 32 (1), 15–29. doi: 10.1007/s13593-011-0027-z

[B48] McAlvay A. C. DiPaola A. D’Andrea A.C. Ruelle M. L. Mosulishvili M. Halstead P.. (2022). Cereal species mixtures: an ancient practice with potential for climate resilience. A review. Agron. Sustain. Dev. 42 (5), 1005. doi: 10.1007/s13593-022-00832-1

[B49] Mead R. Willey R. W. (1980). The concept of a ‘Land equivalent ratio’ and advantages in yields from intercropping. Exp. Agric. 16 (3), 217–228. doi: 10.1017/S0014479700010978

[B50] Miele V. Guill C. Ramos-Jiliberto R. Kéfi S. (2018). The diversity of interaction types drives the functioning of ecological communities. Ecology. 1–18. doi: 10.1101/411249

[B51] Montazeaud G. Violle C. Fréville Hélène Luquet D. Ahmadi N. Courtois B.. (2018). Crop mixtures: does niche complementarity hold for belowground resources? An experimental test using rice genotypic pairs. Plant Soil 424 (1–2), 187–202. doi: 10.1007/s11104-017-3496-2

[B52] Montazeaud G. Violle C. Roumet P. Rocher A. Ecarnot M. Compan Frédéric. (2020). Multifaceted functional diversity for multifaceted crop yield: towards ecological assembly rules for varietal mixtures.” Edited by marney isaac. J. Appl. Ecol. 57 (11), 2285–2955. doi: 10.1111/1365-2664.13735

[B53] Naeem S. Duffy J.E. Zavaleta E. (2012). The functions of biological diversity in an age of extinction. Science 336 (6087), 1401–1406. doi: 10.1126/science.1215855 22700920

[B54] Newton A. C. Skelsey P. (2023). Understanding the effect of component proportions on disease control in two-component cultivar cereal mixtures using a pathogen dispersal scaling hypothesis. Sci. Rep. 13 (1), 40915. doi: 10.1038/s41598-023-31032-w PMC10008548 36906626

[B55] OECD-FAO (2023). OECD-FAO agricultural outlook (OECD Publishing). doi: 10.1787/agr-outl-data-en

[B56] Panda D. Mishra S. S. Behera P. K. (2021). Drought tolerance in rice: focus on recent mechanisms and approaches. Rice Sci. 28 (2), 119–325. doi: 10.1016/j.rsci.2021.01.002

[B57] Pélissier Rémi Buendia L. Brousse A. Temple C. Ballini E. Fort F.. (2021). Plant neighbour-modulated susceptibility to pathogens in intraspecific mixtures. J. Exp. Bot. 72 (18), 6570–6805. doi: 10.1093/jxb/erab277 34125197 PMC8483782

[B58] Qi H. Yang J. Yin C. Zhao J. Ren X. Jia S.. (2019). Analysis of pyricularia oryzae and *P. Grisea* from different hosts based on multilocus phylogeny and pathogenicity associated with host preference in China. Phytopathology® 109 (8), 1433–1405. doi: 10.1094/PHYTO-10-18-0383-R 30973308

[B59] Raboin A. R. Dzido J.-L. Frouin J. Radanielina T. Tharreau D. Dusserre J.. (2013). Upland rice varieties for the highlands of Madagascar: Review of a 25-year-long breeding program. Cahiers Agricultures 22 (5), 450–585. doi: 10.1684/agr.2013.0624

[B60] Raboin T. R. Radanielina T. Ramanantsoanirina A. Ahmadi N. Dusserre J. (2014). Upland rice varieties for smallholder farming in the cold conditions in Madagascar’s tropical highlands. Field Crops Res. 169 (December), 11–20. doi: 10.1016/j.fcr.2014.09.006

[B61] Raboin A. Ramanantsoanirina J. Dusserre F. Razasolofonanahary D. Tharreau C. Lannou. (2012). Two-component cultivar mixtures reduce rice blast epidemics in an upland agrosystem: cultivar mixtures and blast in upland rice. Plant Pathol. 61 (6), 1103–1115. doi: 10.1111/j.1365-3059.2012.02602.x

[B62] Radanielina T. Ramanantsoanirina A. Raboin L.-M. Ahmadi N. (2013). Determinants of rice varietal diversity in the region of Vakinankaratra (Madagascar). Cahiers Agricultures 22 (5), 442–495. doi: 10.1684/agr.2013.0648

[B63] Reiss E. R. Drinkwater L. E. (2018). Cultivar mixtures: A meta-analysis of the effect of intraspecific diversity on crop yield. Ecol. Appl. 28 (1), 62–775. doi: 10.1002/eap.1629 28940830

[B64] Revilla-Molina I. M. (2009). Genetic diversity for sustainable rice blast management in China: adoption and impact 144.

[B65] Richards R. A. (2000). Selectable traits to increase crop photosynthesis and yield of grain crops. J. Exp. Bot. 51 (suppl_1), 447–458. doi: 10.1093/jexbot/51.suppl_1.447 10938853

[B66] Searle (2012). Population marginal means in the linear model: an alternative to least squares means. Am. Statistician 34, 216–221. doi: 10.1080/00031305.1980.10483031

[B67] Singh R. P. Singh P. K. Rutkoski J. Hodson D. P. He X. Jørgensen L. N.. (2016). Disease impact on wheat yield potential and prospects of genetic control. Annu. Rev. Phytopathol. 54 (1), 303–225. doi: 10.1146/annurev-phyto-080615-095835 27296137

[B68] Smithson J. B. Lenné J. M. (1996). Varietal mixtures: A viable strategy for sustainable productivity in subsistence agriculture. Ann. Appl. Biol. 128 (1), 127–158. doi: 10.1111/j.1744-7348.1996.tb07096.x

[B69] Snyder L. D. Gómez M. I. Mudrak E. L. Power A. G. (2021). Landscape-dependent effects of varietal mixtures on insect pest control and implications for farmer profits. Ecol. Appl. 31 (2), 1–11. doi: 10.1002/eap.2246 PMC7988554 33124091

[B70] Snyder L. D. Gómez M. I. Power A. G. (2020). Crop varietal mixtures as a strategy to support insect pest control, yield, economic, and nutritional services. Front. Sustain. Food Syst. 4 (May). doi: 10.3389/fsufs.2020.00060

[B71] Tall H. Lachaux Marlène Diallo A. Wonni I. Tékété C. Verdier Valérie. (2022). Confirmation report of bacterial leaf streak disease of rice caused by xanthomonas oryzae pv. *Oryzicola* in Senegal. Plant Dis. 106 (8), 22535. doi: 10.1094/PDIS-11-21-2481-PDN

[B72] Tilman D. Reich P. B. Knops J. M.H. (2006). Biodiversity and ecosystem stability in a decade-long grassland experiment. Nature 441 (7093), 629–632. doi: 10.1038/nature04742 16738658

[B73] Turnbull L. A. Isbell F. Purves D. W. Loreau M. Hector A. (2016). Understanding the value of plant diversity for ecosystem functioning through niche theory. Proc. R. Soc. B: Biol. Sci. 283 (1844), 20160536. doi: 10.1098/rspb.2016.0536 PMC5204137 27928043

[B74] Wang S. Isbell F. Deng W. Hong P. Dee L. E. Thompson P.. (2021). How complementarity and selection affect the relationship between ecosystem functioning and stability. Ecology 102 (6), 1–12. doi: 10.1002/ecy.3347 33742438

[B75] Weigelt A. Mommer L. Karl A. Iversen C. M. Bergmann J. Bruelheide H.. (2021). An integrated framework of plant form and function: the belowground perspective. New Phytol. 232 (1), 42–59. doi: 10.1111/nph.17590 34197626

[B76] Werf W. v. d. (2021). Comparing performance of crop species mixtures and pure stands. Front. Agric. Sci. Eng. 0 (0), 9. doi: 10.15302/J-FASE-2021413

[B77] Wuest S. E. Peter R. Niklaus P. A. (2021). Ecological and evolutionary approaches to improving crop variety mixtures. Nat. Ecol. Evol. 5 (8), 1068–1775. doi: 10.1038/s41559-021-01497-x 34211140

[B78] Yachi S. Loreau M. (1999). Biodiversity and ecosystem productivity in a fluctuating environment: the insurance hypothesis. Proc. Natl. Acad. Sci. 96 (4), 1463–1685. doi: 10.1073/pnas.96.4.1463 9990046 PMC15485

[B79] Zhang J. L. Rossman A. Y. Aoki T. Chuma I. Crous P. W. Dean R.. (2016). Generic names in magnaporthales. IMA Fungus 7 (1), 155–159. doi: 10.5598/imafungus.2016.07.01.09 27433445 PMC4941683

[B80] Zhang G. Yang Z. Dong S. (2011). Interspecific competitiveness affects the total biomass yield in an alfalfa and corn intercropping system. Field Crops Res. 124 (1), 66–735. doi: 10.1016/j.fcr.2011.06.006

[B81] Zhu Y. Chen H. Fan J. Wang Y. Li Y. Chen J.. (2000). Genetic diversity and disease control in rice. Nature 406 (6797), 718–722. doi: 10.1038/35021046 10963595

[B82] Zhu S. Morel Jean-Benoît (2019). Molecular mechanisms underlying microbial disease control in intercropping. Mol. Plant-Microbe Interactions® 32 (1), 20–245. doi: 10.1094/MPMI-03-18-0058-CR 29996677

