# Supplementary material for: Upland rice varietal mixtures in Madagascar: evaluating the effects of varietal interaction on crop performance
Source: Front Plant Sci. 2023 Nov 20;14:1266704. doi: 10.3389/fpls.2023.1266704 (PMC10694222; doi:10.3389/fpls.2023.1266704)
Supplement: Supplementary file 1 [file DataSheet_1.pdf]

Table S1 : Comparison of soil physical and chemical properties between Site ANK and Site IVO in different horizons

		Physical properties					***Chemical properties***			
		Clay (0 - 2µ) %	*Fine silts (2 - 20µ) %*	*Coarse silt (20 - 50µ) %*	*Fine sand (50 - 200µ) %*	*Coarse sand (0.2 - 2mm) %*	*pH*	*CO (g.kg-1)*	*N tot (g.kg-1)*	*P ass (mg.kg-1)*
**Site ANK**	*Horizon 0 to 10 cm*	38.76	11.54	6.08	9.3	34.32	5.02	21.47	1.72	7.09
	Horizon 10 to 20 cm	45.6	12.23	6.46	9.52	26.2	4.83	20.34	1.58	4.83
	Horizon 20 to 30 cm	55.05	9.09	5.03	7.46	23.36	4.65	14.59	1.29	3.18
**Site IVO**	*Horizon 0 to 10 cm*	36.15	24.75	9.08	9.41	20.61	5.17	17.6	1.44	9.26
	Horizon 10 to 20 cm	39.19	24.37	8.87	8.78	18.8	5.08	16.11	1.3	6.93
	Horizon 20 to 30 cm	46.1	21.97	7.57	7.33	17.04	4.89	15.67	1.16	4.69

Table S2 : Phenological and phenotypic characteristics of the four upland rice varieties. Data extracted from experiment in the site ANK in 2020-2021 (Rahajaharilazaet al. in prep)

		**Agronomic performances**									**Resistance to environmental stresses**				
		Grain Yield	Biomass yield	Harvest Index	Panicle per plant	Spikelet per panicle	Full grain per panicle	Empty grain per panicle	1000 grains weight	Sterility	Resistance to abiotic stress	White worm attack	Weed's biomass	Pyricularia incidence	Pyricularia severity
		(kg.ha-1)	*(kg.ha-1)*	-	*(nb)*	*(nb)*	*(nb)*	*(nb)*	*(g)*	*(%)*	*(score)*	*(%)*	*(g)*	*(%)*	*(cm²)*
**A**	**EARLY_MUTANT_IAC_165**	4 476	5 052	0.45	13	82	68.9	2.7	31.4	15	8.4	10.4	14.4	49.8	3.72
**B**	**F152**	3 553	4 119	0.43	13	64	51.8	2.7	28.7	18	11.2	7.1	6.7	39.6	2.92
**C**	**F154**	3 602	3 993	0.41	12	76	54.0	2.9	32.3	28	11.6	14.3	16.8	48	3.26
**D**	**DOURADO_PRECOCE**	3 553	4 294	0.42	10	81	60.7	3.4	31.0	21	7.4	6.8	28.6	87.6	26

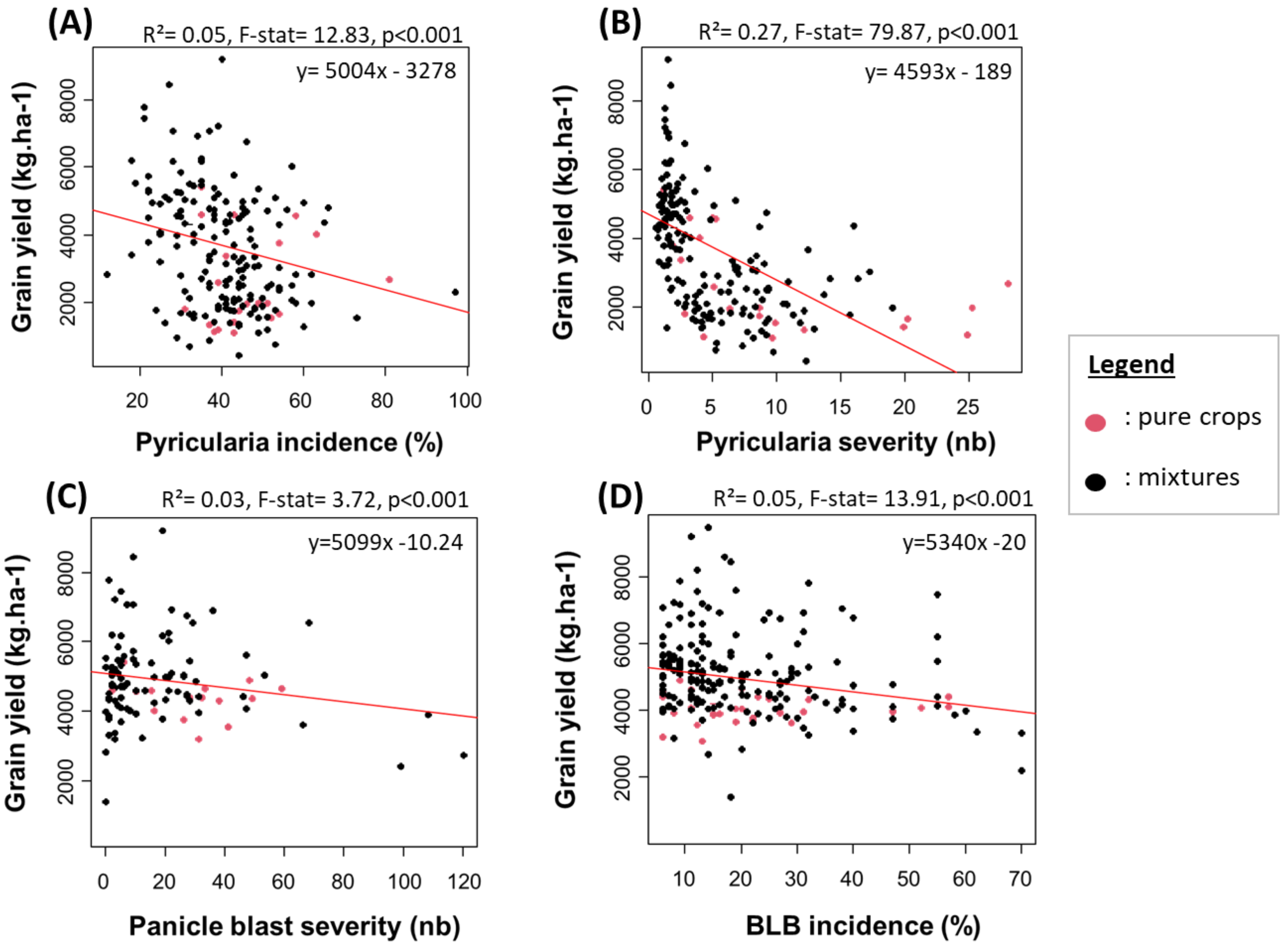

Figure S1:Correlation between grain yield and environmental biotic constraints: (A) blast incidence, (B) blast severity, (C) panicle blast and (D) BLB disease incidence. Dots in red are pure crops and dots in black are plots planted with varietal mixtures.

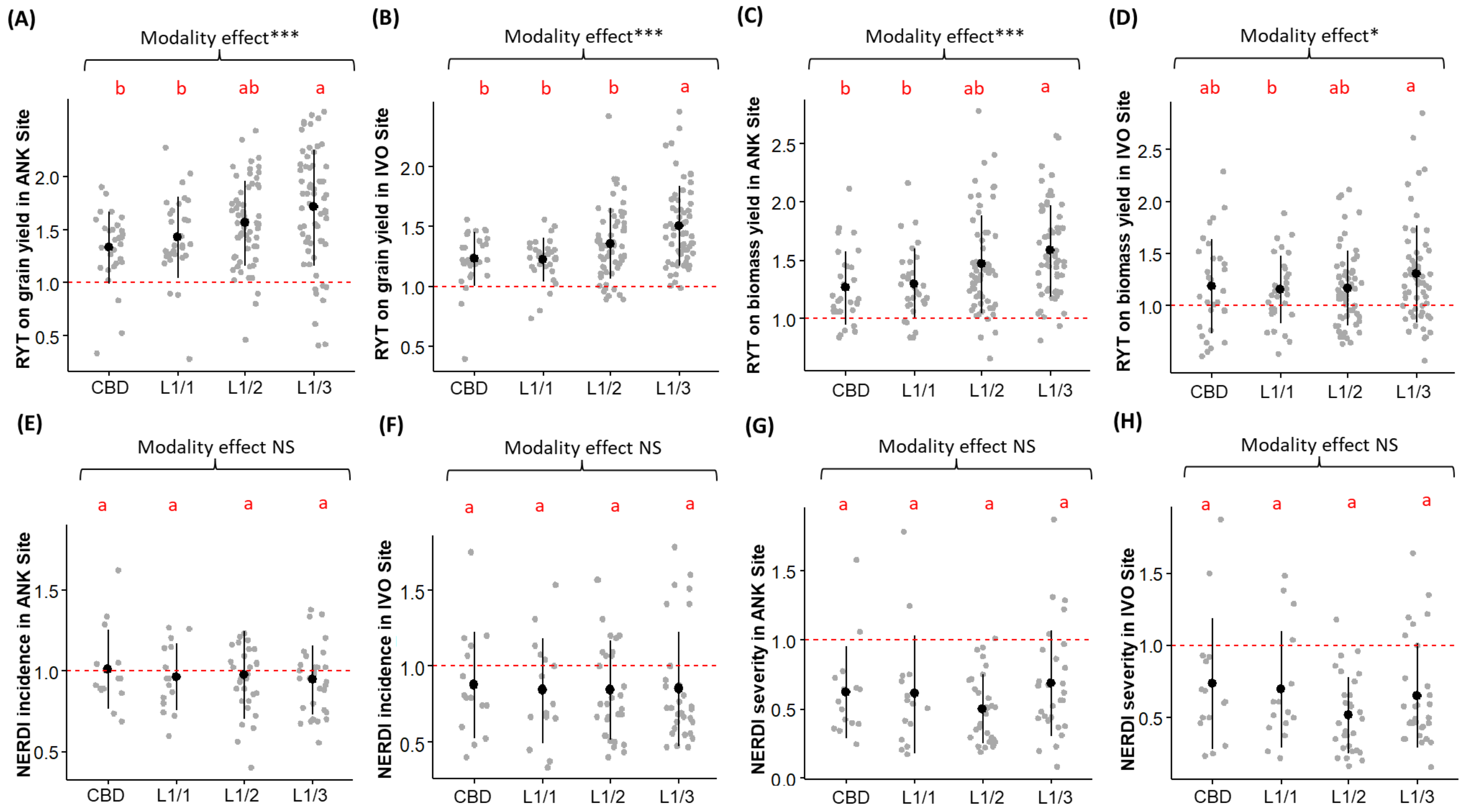

Figure S2: RYT for grain and biomass yield and NERDI values for disease incidence and severity of different spatial arrangements in the two sites. The letters 'a' and 'b' indicate significant differences between arrangements within each sites at the 5% level of confidence (linear model analysis).

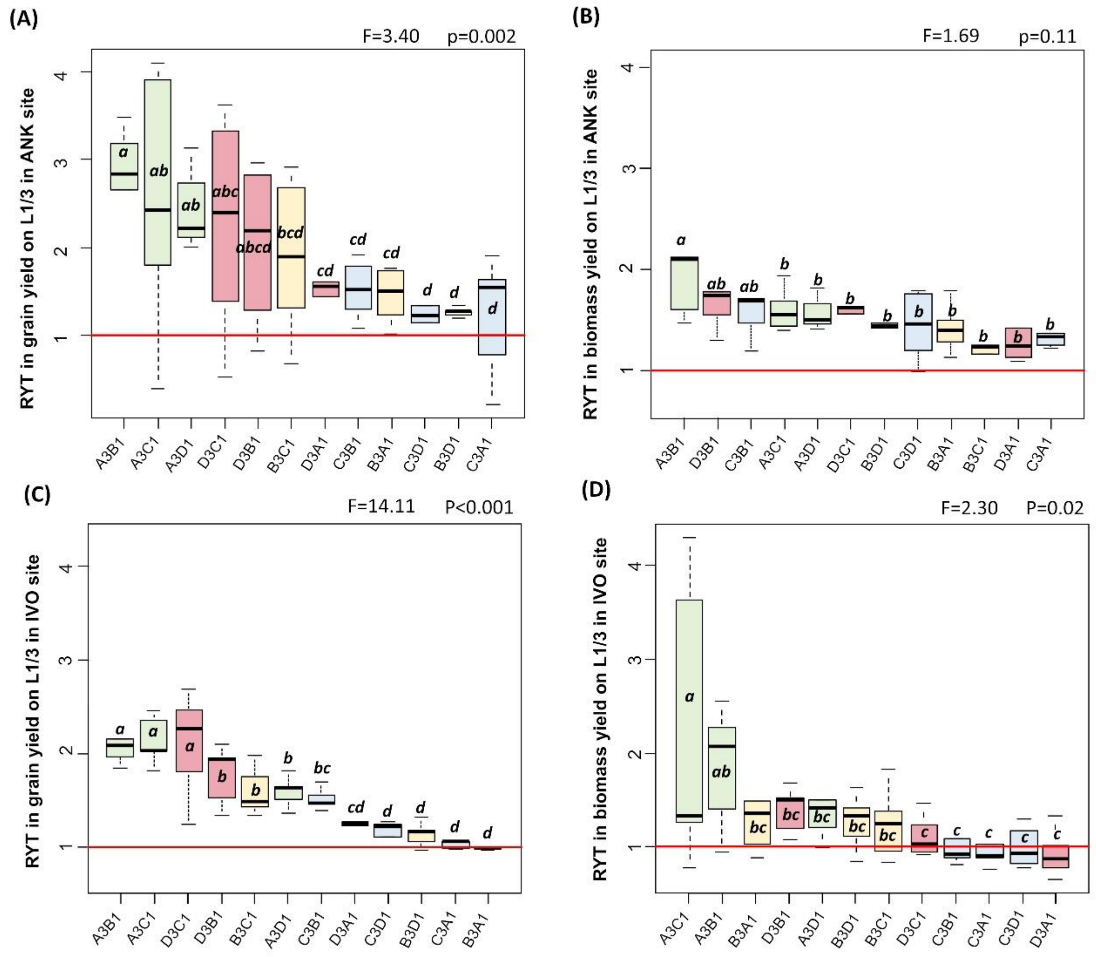

Figure S3: RYT values for grain and biomass yield for L1/3 repartition in the two sites . RYT in ANK site (panels A and B), in IVO site (panels C and D). Mixture effects are assessed using ANOVA and letters 'a' and 'b' indicate significant differences at the 5% level of confidence. The colours indicate the high-dominant varieties in the mixtures (75% for RYT and 63% for RRT). Green for A, yellow for B, blue for C and red for D.
